# Data-Driven Defragmentation: Achieving Value-Based Sarcoma and Rare Cancer Care Through Integrated Care Pathway Mapping

**DOI:** 10.3390/jpm15050203

**Published:** 2025-05-19

**Authors:** Bruno Fuchs, Philip Heesen

**Affiliations:** 1 Sarcoma Center, Department of Orthopaedics and Trauma, LUKS University Hospital, 6000 Lucerne, Switzerland; fuchs@sarcoma.surger; 2 IPU, Department of Orthopaedics and Trauma, LUKS University Hospital, 6000 Lucerne, Switzerland; 3 Faculty of Health Sciences and Medicine, University of Lucerne, 6005 Lucerne, Switzerland; 4 Sarkomzentrum KSW, Klinik für Orthopädie und Traumatologie, Kantonsspital Winterthur, 8400 Winterthur, Switzerland; 5 Medical Faculty, University of Zurich, 8032 Zurich, Switzerland

**Keywords:** value-based healthcare, Sarcoma, Artificial Intelligence, data

## Abstract

Sarcomas, a rare and complex group of cancers, require multidisciplinary care across multiple healthcare settings, often leading to delays, redundant testing, and fragmented data. This fragmented care landscape obstructs the implementation of Value-Based Healthcare (VBHC), where care efficiency is tied to measurable patient outcomes.ShapeHub, an interoperable digital platform, aims to streamline sarcoma care by centralizing patient data across providers, akin to a logistics system tracking an item through each stage of delivery. ShapeHub integrates diagnostics, treatment records, and specialist consultations into a unified dataset accessible to all care providers, enabling timely decision-making and reducing diagnostic delays. In a case study within the Swiss Sarcoma Network, ShapeHub has shown substantial impact, improving diagnostic pathways, reducing unplanned surgeries, and optimizing radiotherapy protocols. Through AI-driven natural language processing, Fast Healthcare Interoperability Resources, and Health Information Exchanges, HIEs, the platform transforms unstructured records into real-time, actionable insights, enhancing multidisciplinary collaboration and clinical outcomes. By identifying redundancies, ShapeHub also contributes to cost efficiency, benchmarking treatment costs across institutions and optimizing care pathways. This data-driven approach creates a foundation for precision medicine applications, including digital twin technology, to predict treatment responses and personalize care plans. ShapeHub offers a scalable model for managing rare cancers and complex diseases, harmonizing care pathways, improving precision oncology, and transforming VBHC into a reality. This article outlines the potential of ShapeHub to overcome fragmented data barriers and improve patient-centered care.

## 1. Introduction

Sarcomas, as a category of rare cancers, present significant clinical and logistical challenges due to their heterogeneous nature and low incidence rates [1]. Diagnosing and managing sarcomas often involves multiple healthcare settings, from primary care and community hospitals to specialized cancer centers [2]. This multi-step process results in fragmented care pathways that not only delay diagnosis but also lead to data being scattered across various institutions [2]. This fragmented data landscape, or “data vortex”, hinders the implementation of personalized medicine and Value-Based Healthcare (VBHC), where efficiency and quality of care are intrinsically linked to measurable patient outcomes.

Our proposed solution is a data-driven defragmentation approach facilitated through an integrated interoperable digital platform called ShapeHub. ShapeHub is designed to organize care around and across specific medical conditions, akin to the logistics system that tracks an item through various stages of delivery. For example, ShapeHub ensures that all relevant patient data—such as diagnostic results, treatment updates, and specialist consultations—are captured and shared across care providers, reducing delays and enhancing continuity of care. This ensures that the patient’s journey through different healthcare settings is streamlined and transparent, similar to how logistics systems manage the transport of goods. This platform centralizes and harmonizes patient information across healthcare sites, enabling continuous and cohesive care pathways. By ensuring that every encounter—whether a simple diagnostic procedure or a complex surgical intervention—contributes to a unified dataset, ShapeHub creates a cohesive and comprehensive as well as automatically created record. This transforms the fragmented care model into a streamlined, value-oriented system. This harmonization not only improves workflow efficiency but enables personalized treatment decisions by leveraging artificial intelligence (AI)-powered risk stratification and precision oncology tools. This article explores how this model works in practice, offering a roadmap for optimizing sarcoma care and care for other rare cancers.

## 2. Theoretical and Practical Background

### 2.1. Fragmentation as a Systemic Challenge

Fragmentation in sarcoma care is multifaceted. Patients are often first seen by their family or primary care physician, who may initiate initial diagnostics before referring them to specialists [3]. Patients subsequently undergo numerous diagnostics—imaging, biopsies, and lab tests—at different institutions before reaching a definitive diagnosis [3]. Additionally, treatment pathways involve a range of specialists, including oncologists, surgeons, radiologists, and pathologists [4]. Each point of care generates data that remain isolated within the institution’s siloed system. This data vortex prevents healthcare providers from having a comprehensive view of the patient’s journey, resulting in repeated tests, miscommunications, and delays that negatively impact outcomes.

### 2.2. Digital Interoperable Platforms as Enablers

Digital interoperable platforms like ShapeHub aim to address these challenges by acting as a centralized hub that will integrate data from all care points, including diagnostic imaging, treatment records, specialist consultations, and follow-up care. First, access to the platform is facilitated through a trackable patient identification system based on World ID, which functions similarly to a logistics tracking system—like those used in supply chain management to ensure items are transported seamlessly [5]. In healthcare, each patient interaction can be efficiently tracked across all care settings, enhancing both transparency and the quality of care.

After access is granted, it is crucial to harmonize data acquisition using the principles of click and collect, talk and capture (i.e., Natural Language Processing (NLP)), and scan and sync. The latter includes an AI ontology algorithm that screens for parameters from PDF documents provided by other institutions [6]. ShapeHub’s internal data collection is designed to be directly analyzable, minimizing the need for external standard conversions.

By utilizing interoperability standards such as FHIRs (Fast Healthcare Interoperability Resources) [7] and leveraging technologies like Health Information Exchanges (HIEs) [8,9], blockchain, and a trackable system for patient interactions, ShapeHub is planned to facilitate the seamless transfer of information between healthcare providers, ensuring that every patient interaction is recorded and accessible for one given medical condition and its associated comorbidities. FHIRs and HIEs are complementary tools that support efficient data exchange, with FHIRs providing the standard for data formats and HIEs facilitating the actual data exchange. Furthermore, the platform will employ AI algorithms and NLP to harmonize and structure data, transforming previously unstructured information into actionable insights.

ShapeHub not only reduces redundancies but also ensures that all healthcare providers involved have access to up-to-date, accurate information, creating a unified patient journey that supports VBHC. This comprehensive access to integrated patient data directly contributes to better clinical decision-making, improved patient outcomes, and enhanced quality of care. Furthermore, ShapeHub facilitates personalized treatment recommendations and dynamic treatment adjustments based on evolving clinical and molecular data and by continuously integrating real-time patient responses.

As the volume of healthcare data continues to grow, AI and machine learning (ML) offer transformative potential for optimizing sarcoma care. By leveraging AI-driven analytics, ShapeHub can enable dynamic risk stratification, predictive modeling, and decision support, enhancing the precision and efficiency of sarcoma management. Through natural language processing (NLP), unstructured radiology, pathology, and clinical reports can be systematically processed, extracting key diagnostic and prognostic insights in real time [10]. Additionally, machine learning models trained on multi-omics data (genomics, proteomics, imaging, and clinical histories) could refine personalized treatment strategies by predicting treatment responses, toxicities, and long-term outcomes [11].

An essential component of AI integration within ShapeHub is the use of federated learning, which allows multi-institutional collaboration without directly sharing patient data [12]. This decentralized approach ensures compliance with data privacy regulations such as GDPR while enabling continuous model refinement across multiple centers. By systematically incorporating real-world-time feedback loops, AI models embedded within ShapeHub can adapt to evolving clinical knowledge, offering tailored treatment recommendations that align with precision oncology principles.

Beyond immediate clinical benefits, AI-powered insights also contribute to long-term outcome tracking and real-world validation. The ability to conduct real-time comparative effectiveness research will allow for the continuous evaluation of treatment pathways [13]. Target trial emulation, a method used within ShapeHub, provides an innovative way to emulate randomized controlled trials (RCTs) by retrospectively comparing different treatment strategies across patient cohorts [14]. This allows for the identification of best practices and the optimization of referral pathways, ultimately reducing unnecessary interventions such as unplanned “whoops” surgeries.

To ensure the sustainable impact of AI-driven decision support, it is crucial to establish standardized validation frameworks that assess ShapeHub’s effectiveness across different healthcare settings. By benchmarking patient outcomes and costs over time, we can systematically assess how digital tools improve clinical efficiency, patient experience, and healthcare resource utilization. Future directions should focus on expanding ShapeHub’s capabilities to include predictive digital twin technology, wherein virtual patient simulations can help forecast treatment outcomes before interventions are initiated [15]. This will facilitate truly personalized medicine, where therapy adjustments are continuously optimized based on real-time patient data.

The integration of AI and long-term validation methodologies within ShapeHub represents a paradigm shift in rare cancer management. By transforming fragmented, siloed data into actionable insights, ShapeHub lays the foundation for a learning healthcare system that continuously evolves, improving patient care and advancing VBHC principles.

### 2.3. Related Work: Positioning ShapeHub Among Existing Digital Care Pathways

Several digital platforms and frameworks have attempted to address the problem of care fragmentation and interoperability in oncology and rare disease management. While these initiatives have made progress in standardizing health data or supporting multidisciplinary collaboration, ShapeHub represents a unique synthesis of these efforts, integrating real-time data harmonization, AI-driven analytics, and care pathway mapping within a single platform.

#### 2.3.1. Integrated Practice Units and Value-Based Healthcare

The concept of organizing care around specific medical conditions, as pioneered by Porter and Teisberg’s VBHC framework [16], has led to the development of Integrated Practice Units (IPUs) in institutions like the Cleveland Clinic and MD Anderson Cancer Center. These units collocate multidisciplinary teams to streamline decision-making and measure outcomes. However, most IPUs rely on co-location rather than digital infrastructure, limiting their scalability across fragmented systems or networks. ShapeHub expands on the IPU model by enabling virtual integration through data, not geography.

#### 2.3.2. Electronic Health Record (EHR) Enhancements and Learning Health Systems

Platforms such as Epic Cosmos, Flatiron Health, and ASCO’s CancerLinQ have attempted to extract structured insights from electronic health records (EHRs) in oncology. For example, Flatiron’s oncology-specific EHR offers real-world evidence from de-identified datasets across many US practices. However, these systems often function within a single-vendor ecosystem and are not purpose-built for rare diseases or real-time clinical decision-making [17]. In contrast, ShapeHub prioritizes interoperability across institutions and care settings, and its modular, disease-agnostic design makes it suitable for rare and complex conditions.

#### 2.3.3. National Interoperability Initiatives and Health Information Exchanges (HIEs)

Countries such as France, the UK, and the Netherlands have deployed national health data platforms like the Dossier Médical Partagé (DMP) or MedMij, aiming to standardize longitudinal health records [18,19]. These platforms primarily serve administrative or patient communication functions and often struggle with deep clinical integration, particularly in oncology. Similarly, traditional HIEs facilitate data sharing but do not provide the structured care pathway analytics, AI integration, or decision support features built into ShapeHub.

#### 2.3.4. Rare Disease Networks and European Reference Networks (ERNs)

The European Reference Networks (ERNs) for rare diseases promote cross-border collaboration among centers of expertise. While ERNs focus on knowledge exchange and remote consultation, they currently lack a shared, structured, interoperable data platform tailored to clinical decision-making [20]. ShapeHub can complement these networks by offering a technical solution for harmonized care tracking and pathway optimization across borders.

#### 2.3.5. AI-Driven Clinical Decision Support Systems

Recent efforts to incorporate AI into oncology include IBM Watson for Oncology and several NLP tools for radiology and pathology interpretation [21]. However, such tools are typically siloed, addressing only one modality or clinical question. ShapeHub’s integration of AI, natural language processing, and federated learning within a unified care pathway framework enables not just diagnostic support but end-to-end care optimization across diagnostics, surgery, radiotherapy, and follow-up.

In summary, while prior systems have addressed components of data integration, outcome benchmarking, or digital collaboration, ShapeHub uniquely combines them into a real-time, interoperable, and AI-enhanced infrastructure specifically built for rare and complex diseases like sarcoma. It thus serves as a bridge between visionary concepts (VBHC, learning health systems, and digital twins) and practical, implementable solutions.

## 3. Case Study: ShapeHub’s Implementation in the Swiss Sarcoma Network (SSN)

**Figure 1:**
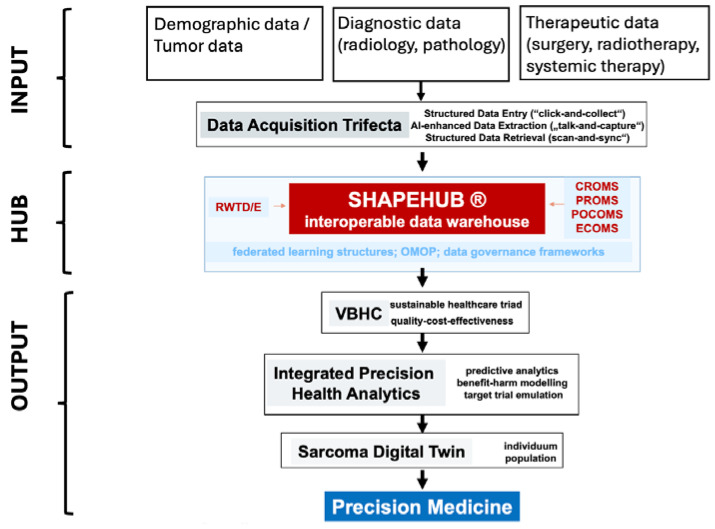
Overview of input and output of ShapeHub. CROM: Clinician Report Outcome Measures; PROM: Patient Reported Outcome Measures; POCOMS: Patient Oriented and Centric Outcome Measures; ECOMS: Economic Outcome Measures; RWTD/E: Real World Time Data/Evidence; VBHC: Valued Based Healthcare.

The Swiss Sarcoma Network (SSN) provides an illustrative example of how the current version of ShapeHub has already made significant contributions through harmonized structured data integration. Figure 1 presents an overview of the input and output of ShapeHub. Specifically, ShapeHub has improved diagnostic pathways, reduced unnecessary surgeries, and optimized treatment protocols, leading to better care outcomes. Here, we delve into three specific scenarios that highlight the digital interoperable platform’s potential impact.

### 3.1. Example 1: Refining Diagnostic Pathways

Traditionally, sarcoma patients experience diagnostic delays, often undergoing multiple scans and consultations before a diagnosis is confirmed [3]. Within the SSN, a recent real-world-time analysis included 1028 patients presented to the multidisciplinary tumor board between 2018 and 2021.

The study revealed that the patient interval (a median of 2.3 months) contributed the largest share to the total diagnostic delay, followed closely by the secondary care interval, which represented the most significant component of the diagnostic interval itself. Notably, older patient age, axial tumor localization, and larger tumor size were identified as factors associated with longer intervals and an increased probability of sarcoma.

Specifically, sarcomas localized to the axial skeleton had a statistically significant longer diagnostic delay compared to those in the extremities (median 6.7 vs. 4.1 months). Through ShapeHub’s real-time tracking of referral steps and delays, these findings allow for the dynamic identification of bottlenecks and propose actionable measures, such as reducing secondary care waiting times, to streamline the diagnostic journey and improve early detection rates in sarcoma patients.

### 3.2. Example 2: Reducing Unplanned “Whoops” Surgeries

Unplanned “whoops” surgeries occur when tumor removal is attempted without full staging or multidisciplinary input and without expecting the diagnosis of sarcoma. ShapeHub aims to minimize this risk by consolidating patient information from local hospitals, imaging centers, and sarcoma specialists into a single view. By providing specialists with comprehensive patient records, we believe that surgical decisions will be based on a complete and accurate understanding of the tumor’s characteristics and the patient’s overall health. ShapeHub has already contributed significantly to understanding the reasons for “whoops” surgeries and their consequences. Additionally, ShapeHub has enabled target trial emulation (TTE) to compare groups in analogy to randomized controlled trials (RCTs), offering valuable insights into the impact of different referral and treatment pathways [22]. As part of ShapeHub, TTE can be used for any possible comparison of patient groups, providing a flexible tool for evaluating outcomes across various aspects of care. The study demonstrated that 19.6% of all patients experienced an unplanned excision (UE), and these patients had a 2.27-fold increased risk (95% CI, 1.12–4.60) of local recurrence compared to those undergoing planned resections.

Interestingly, despite the increased risk of local failure, no statistically significant differences were observed in metastasis-free survival, cancer-specific survival, or overall survival between the two groups during the follow-up period. Furthermore, the presence of residual tumor after UE was observed in 31% to 74% of cases, which substantially contributed to the observed risk of local recurrence. By offering an integrated patient overview before any surgical intervention, ShapeHub supports the early identification of suspicious lesions, facilitates timely referral to specialized centers, and thus significantly reduces the likelihood of unplanned surgeries and their associated adverse outcomes.

### 3.3. Example 3: Optimizing Radiotherapy and Surgical Protocols

In sarcoma care, the timing and coordination of treatments, such as radiotherapy and surgery, are critical. We employed a different radiotherapy (RT) schedule, specifically an ultrahypofractionated approach using only 5 fractions of 5 Gy each, compared to the traditional 25 fractions of 2 Gy. The median interval between radiation completion and surgery was 16 days, substantially shorter than traditional approaches, which often recommend waiting periods of 4–8 weeks. Additionally, clear resection margins (R0) were achieved in 92% of patients, and early treatment-related toxicity was limited to grade 0–1 dermatitis. Through real-time data integration and multidisciplinary scheduling enabled by ShapeHub, these outcomes demonstrate how optimized radiotherapy protocols can be safely accelerated, improving patient experience while maintaining excellent oncologic results. Using ShapeHub, we were able to analyze and compare the outcomes, demonstrating that the shorter schedule provides the same local control rate after 2 years and, importantly, similar low wound healing complication rates as the normofractionated schedule [23]. This highlights the strength of our ShapeHub system, which allows for a real-world-time assessment and comparison of treatment protocols, including the ability to compare results from different institutions and settings prospectively, as well as comparisons across different care groups such as surgical groups, chemotherapy groups, and other care modalities, providing a consistent and standardized approach to evaluating outcomes. The ability to implement an RT schedule that reduces the treatment effort by 80%, while maintaining clinical benefits, significantly lowers operational costs and reduces the burden on patients, who only need to attend 5 sessions instead of 25.

## 4. Addressing Fragmentation: Care Pathway Mapping as a Solution to the Data Vortex

### 4.1. Care Pathway Mapping

ShapeHub’s core function is to map and organize patient journeys from the initial point of care to follow-up. The platform captures each patient encounter—whether at a community hospital, imaging center, or specialist clinic—and integrates it into the central system using a structured data capture approach right from the beginning, ensuring that all data collected are consistent and directly analyzable. This internal data structure minimizes the need for external standard conversions such as FHIRs [7] or OMOP for internal use. However, to ensure collaboration with external systems and institutions, ShapeHub utilizes FHIRs [7] for standardized data formats and HIEs [8,24] to facilitate the actual data exchange, alongside middleware solutions that facilitate communication and data management between healthcare systems. Middleware solutions, analogous to logistics tracking technologies like GS1, facilitate seamless communication between different healthcare systems, ensuring that the data move accurately and efficiently between institutions, just as logistics systems manage the flow of items through complex networks. This ensures that all data related to the patient’s sarcoma care are structured and harmonized, creating a cohesive, real-world-time view of the patient’s health status.

### 4.2. Implementing Solutions to the Data Vortex

- HIE Integration: Through HIEs [8,9], ShapeHub aims to standardize patient information exchange, ensuring that data are consolidated under a single patient record. This integration is expected to eliminate inconsistencies, reduce the duplication of tests, and improve overall care efficiency.
- AI and Blockchain Technologies: AI algorithms will structure data into actionable formats, while blockchain technology is planned to secure and authenticate each data entry, ensuring that information integrity is maintained across all points of care. Patient identification will be managed through systems like World ID [5], functioning as a trackable identification system that securely links patient data across institutions, ensuring that information is accessible to authorized healthcare providers and allowing for efficient tracking throughout the care pathway.

By employing these methods, ShapeHub seeks to dismantle the data vortex, transforming fragmented records into a unified dataset that healthcare providers can rely on for accurate, evidence-based decision-making.

## 5. Transdisciplinary Collaboration: The Key to Overcoming the Data Vortex

### 5.1. Unified Protocols and Shared Data

One of the most significant goals of ShapeHub is to facilitate transdisciplinary collaboration. Sarcoma care inherently involves multiple specialties, and without a system that integrates and shares data efficiently, care teams often operate in silos. ShapeHub is designed to unify these teams by providing access to a centralized patient record that is updated in real-world time, ensuring that each specialist—whether an oncologist, surgeon, or radiologist—has the most current and complete information.

### 5.2. Impact on Clinical Decision-Making

We demonstrated ShapeHub’s ability to enhance multidisciplinary meetings within SSNs. Specialists are able to review and discuss the same comprehensive dataset, leading to improved treatment planning and coordination. Our approach showed better outcomes in various aspects, reducing variation and improving consistency in care delivery, as evidenced by recent publications.

## 6. Economic Impact and Cost Efficiency

### 6.1. Comprehensive Cost Mapping

One of the key objectives of VBHC is aligning patient outcomes with costs, where cost mapping is synonymous with aligning reimbursement with value [25]. ShapeHub is being designed to enable cost mapping by integrating financial data with clinical outcomes across the patient’s care pathway. We are currently in the stage of defining cost drivers to generate a comprehensive view of treatment costs, distinguishing between cost expenses and cost revenues, while aligning these metrics with the overall value provided to patients.

### 6.2. Benchmarking and Efficiency Gains

Preliminary analyses are exploring how ShapeHub can be used to benchmark costs across different hospitals and treatment modalities. Our focus is on mapping cost expenses per discipline. We have found that the costs of surgeries themselves are consistent across hospitals; however, the overhead costs associated with hospitalization differ significantly. It is not the surgery itself but rather the overhead costs in the context of hospitalization that surpass the expenses of surgery alone. By minimizing duplicate imaging and aligning diagnostic timelines, ShapeHub could help identify these discrepancies and create opportunities for substantial cost savings while maintaining high-quality outcomes.

## 7. Future Vision: Precision Medicine and Digital Twins

### 7.1. The Role of Digital Twins

As ShapeHub consolidates and harmonizes care pathways, it lays the groundwork for advanced care models like digital twins—virtual representations of patient health [26]. These models require high-quality, structured data, which ShapeHub aims to provide through its integrated approach. We are developing the infrastructure needed to simulate digital twins for sarcoma patients, with the goal of predicting treatment responses and optimizing care plans.

### 7.2. Scalability Beyond Sarcoma

The architecture of ShapeHub is designed to be scalable to other rare and complex cancers. Although currently focused on sarcoma care, the platform’s algorithms and data modules are being developed to support a broader range of conditions. We expect that the success seen in sarcoma care can be expanded to other disease areas in the future.

## 8. Implications

The development and implementation of ShapeHub carry significant implications beyond sarcoma care, offering a model for transforming how rare and complex diseases are managed in modern healthcare systems.

### 8.1. Health System Integration and Learning Health Systems

By structuring and harmonizing patient data across institutions, ShapeHub aligns closely with the vision of a learning health system—a healthcare model where data and experience are continuously analyzed and fed back to improve care quality and efficiency in real time [27]. The ability to incorporate real-world-time patient feedback, perform target trial emulation, and continuously update clinical pathways supports adaptive care models that can evolve with emerging evidence.

### 8.2. Policy and Reimbursement Models Aligned with VBHC

The integration of clinical and financial data enables precise cost–outcome mapping, an essential requirement for implementing Value-Based Healthcare (VBHC) at scale. This supports a transition away from fee-for-service models toward outcome-based reimbursement structures, as championed by policymakers in the EU and the U.S. Centers for Medicare & Medicaid Services (CMS) [25]. ShapeHub’s transparent data architecture may also facilitate performance-based contracts and bundled payments for rare cancers.

### 8.3. Clinical Standardization Across Decentralized Networks

Rare cancer management often suffers from variability in care quality across centers. ShapeHub’s structured data architecture enables protocol standardization and benchmarking, reducing unwarranted variation—a key aim in complex oncology pathways [2]. It thus supports networks like European Reference Networks (ERNs) for rare diseases, which rely heavily on shared decision-making and real-time data sharing across national borders [20].

### 8.4. Enhancing Patient Empowerment and Safety

Access to integrated and up-to-date records empowers patients by reducing redundant procedures and ensuring transparency across transitions of care. In addition, by proactively identifying risk factors for adverse events (e.g., “whoops” surgeries), ShapeHub improves safety and minimizes preventable harm, aligning with global patient safety strategies [28].

### 8.5. Scalable Infrastructure for Digital Twin and Precision Medicine

As digital twins and AI-based precision tools advance, they require structured, longitudinal, and harmonized datasets—precisely the kind of data environment ShapeHub facilitates. This positions it as foundational infrastructure for future-ready, simulation-based treatment planning [15]. Moreover, its modular architecture can be scaled to support other rare and complex diseases, offering a reusable blueprint for digital transformation.

## 9. Conclusions: Call to Action

To implement VBHC effectively in sarcoma care and beyond, healthcare systems must adopt data harmonization strategies to defragment care. ShapeHub demonstrates this by creating a unified, interoperable, and patient-centered platform; care pathways can be transformed, leading to improved outcomes, cost efficiency, and the elimination of data silos. Moreover, the dynamic personalization of therapy and care pathways can be achieved by allowing for the continuous response feedback of clinical data.

We call on healthcare institutions, technology providers, and policymakers to collaborate in developing and scaling such systems. By doing so, we can build a cohesive and sustainable healthcare ecosystem that supports not only sarcoma care but also a wide range of rare and complex diseases.

## Data Availability

No new data were created or analyzed in this study. Data sharing is not applicable to this article.
